# The tumor micro-environment in pediatric glioma: friend or foe?

**DOI:** 10.3389/fimmu.2023.1227126

**Published:** 2023-10-13

**Authors:** Julie Messiaen, Sandra A. Jacobs, Frederik De Smet

**Affiliations:** ^1^Department of Pediatrics, University Hospitals Leuven, Leuven, Belgium; ^2^Laboratory for Precision Cancer Medicine, Translational Cell and Tissue Research, Department of Imaging and Pathology, KU Leuven, Leuven, Belgium; ^3^Pediatric Oncology, Department of Oncology, KU Leuven, Leuven, Belgium

**Keywords:** pediatric glioma, tumor micro-environment, T-cells, myeloid cells, immunotherapy

## Abstract

Brain tumors are the leading cause of morbidity and mortality related to cancer in children, where high-grade glioma harbor the worst prognosis. It has become obvious that pediatric glioma differs significantly from their adult counterparts, rendering extrapolations difficult. Curative options for several types of glioma are lacking, albeit ongoing research efforts and clinical trials. As already proven in the past, inter- and intratumoral heterogeneity plays an important role in the resistance to therapy and thus implicates morbidity and mortality for these patients. However, while less studied, the tumor micro-environment (TME) adds another level of heterogeneity. Knowledge gaps exist on how the TME interacts with the tumor cells and how the location of the various cell types in the TME influences tumor growth and the response to treatment. Some studies identified the presence of several (immune) cell types as prognostic factors, but often lack a deeper understanding of the underlying mechanisms, possibly leading to contradictory findings. Although the TME in pediatric glioma is regarded as “cold”, several treatment options are emerging, with the TME being the primary target of treatment. Therefore, it is crucial to study the TME of pediatric glioma, so that the interactions between TME, tumoral cells and therapeutics can be better understood before, during and after treatment. In this review, we provide an overview of the available insights into the composition and role of the TME across different types of pediatric glioma. Moreover, where possible, we provide a framework on how a particular TME may influence responses to conventional- and/or immunotherapy.

## 1 Introduction

Brain tumors are the leading cause of morbidity and mortality related to cancer in children (1). Within this group of tumors, glioma harbor the worst prognosis (2). The pediatric low-grade glioma (pLGG, grade I and II glioma), have a favorable prognosis, with a 10-year overall survival (OS) of 94% (3), while pediatric high-grade glioma (pHGG; grade III and IV tumors) still have a dismal prognosis, with a median OS of 9 to 15 months (4–6).

Over the years, it has become increasingly obvious that pediatric glioma differ significantly from their adult counterparts, illustrating the necessity to study these tumors as separate entities (5). These pediatric tumors harbor significant differences on both the genetic and epigenetic level, although they can have similar histological appearances, rendering extrapolations from adult studies obsolete (7, 8). In the last years, pediatric brain tumors have been regarded increasingly as a neurodevelopmental disease (9, 10). For instance, the H3.3 K27M-mutant high-grade glioma (HGG), are considered to originate from a pontine glial-committed neural progenitor, where the differentiation along the glial lineage is hampered by this mutation (10).

The tumor micro-environment (TME) includes the non-cancerous cells in and surrounding the tumor such as the immune cells, endothelial cells, microglia, astrocytes and neurons. The TME thus adds another level of heterogeneity between these tumors. In the past, the TME of mainly the adult tumors were studied, and only recently, the focus has turned to their pediatric counterparts. However, studies regarding the TME in pediatric glioma are still sparse. An additional level of complexity resides in the maturation of the immune system during childhood, therefore, caution is needed before extrapolating findings regarding the TME from adult brain tumors to the pediatric types (11).

The major immune infiltrates in pediatric glioma are composed of macrophages and T-cells (12). Brain tumors, especially those of a pediatric origin, have long been regarded as immunologically ‘cold’. These tumors generally have a low mutational burden, with a paucity of neo-antigens. For example, diffuse midline glioma (DMG) and more specifically diffuse intrinsic pontine glioma (DIPG), have been considered as ‘immune cold’. However, in a recent study using transcriptional profiling, DMG had a greater inflammatory TME compared to pediatric HGG occurring in the hemispheres (13). Furthermore, when comparing pediatric glioma based on their immune-scores, it was shown that patients who were identified as “immune-hot” had a longer OS compared with the “immune-cold” tumors (12).

The technologies to study the TME have also evolved, ranging from basic histological analysis to methylation studies and more recently single-cell (dissociated or spatially resolved) studies, although the latter still remain sparse. Recently, a large methylation study on bulk tumor samples by Grabovska et al. identified three types of immune clusters in pediatric brain tumors and found that the average infiltration of immune cells decreased with a higher WHO grade of the tumor (14). However, a lot of information is still lacking, with mainly knowledge gaps on how the TME interacts with the tumor cells and how the location of the different cell types in the tissue context influences the tumor and its response to treatment. With the observation of spatial and temporal heterogeneity of mutations in DIPG (15), we could hypothesize that this evolution might also influence the TME.

Glioma in children are classically treated with a combination of surgery, radiotherapy and/or chemotherapy, depending on the subtype of glioma. However, these treatment modalities can still be associated with high levels of morbidity (16). In the last couple of years, more targeted therapy has been developed, from which some are already being used in the clinical setting e.g. MEK-inhibitors in pLGG such as trametinib (17), while others are still in clinical trial. Additionally, several types of immunotherapy are quickly arising, raising the question about their possible effects and usefulness in the treatment of these tumors. The problems of treating these tumors can partially be explained by the heterogeneity present in these tumors, but possibly the TME plays a role in the response to therapy. To have a better understanding of which patients might benefit from therapies based on immunological principles, it is a necessity to better understand the TME.

In this review, we provide an overview of the available insights into the composition and role of the TME across different types of pediatric glioma. Where possible, we provide a framework on how a particular TME may influence responses to conventional- and/or immunotherapy. In the first part, we provide a comprehensive overview of the different immune and neural cell types present in the TME across different types of pediatric glioma. Subsequently, the effects of the TME on various therapeutic modalities and *vice versa* are discussed.

## 2 The immune microenvironment in pediatric glioma

**Figure 1:**
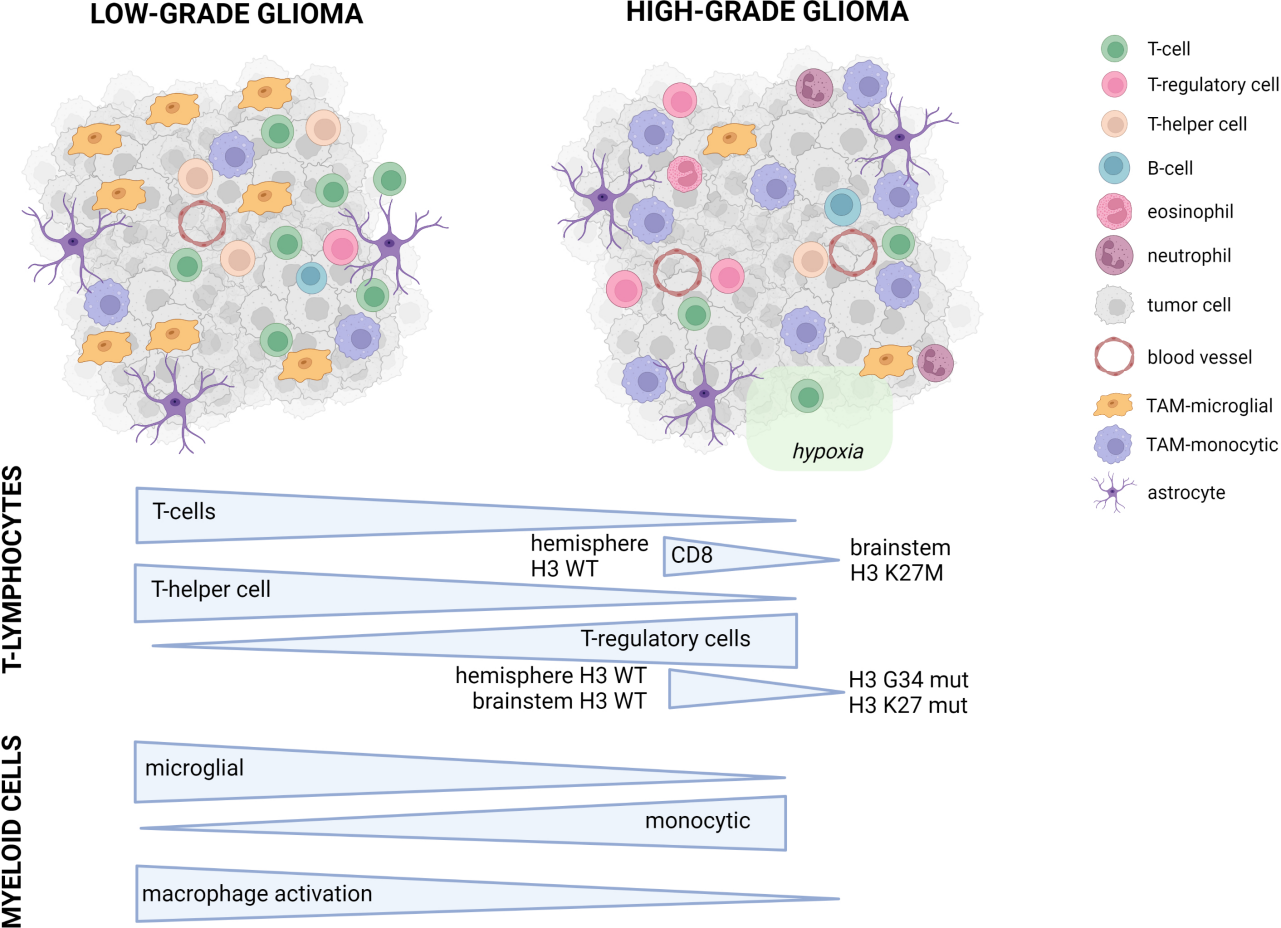
Conceptual overview of the main differences between pediatric LGG and HGG. Symbols used in the figure are explained on the right. In the lower part of the figure, the major differences between pLGG and pHGG for both T-lymphocytes and myeloid cells are illustrated.

Overall, the total amount of infiltrating immune cells in pediatric glioma is limited. Relatively speaking, the amount of immune infiltrates is higher in LGG compared to HGG, with the majority of infiltrating cells being macrophages and T-cells (12, 14), to which we will provide the most attention. In the next subsections, we describe the different types of immune cells from both the innate and adaptive immune system present in the TME. **Figure 1** includes a general overview of the major differences. **Table 1** provides a more detailed overview on the differences between innate and adaptive immunity in pLGG and pHGG.

**Table 1:** Summary of the effects of the cells in the TME on pediatric glioma.

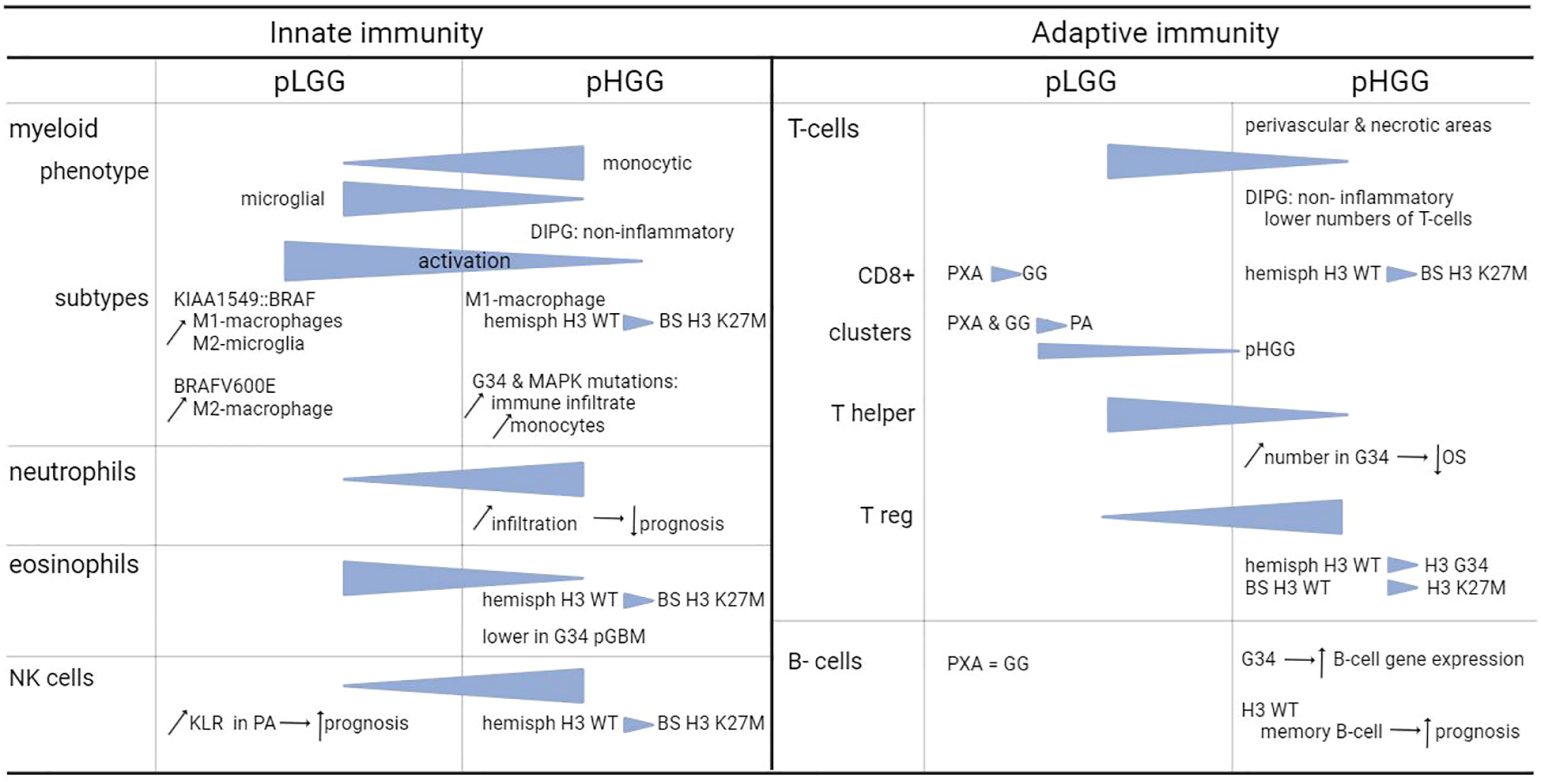	

BS, brainstem; WT, wild-type.

### 2.1 Cells from the innate immune system in the TME

#### 2.1.1 Myeloid cells: macrophages and microglia

Macrophages are one of the major immune infiltrates in pediatric glioma (12).Tumor-associated myeloid (TAM) cells are generally separated into bone-marrow derived macrophages (BMDM) or tumor-associated macrophages from a monocytic origin (moTAM) and macrophages from a microglial origin (mgTAM). In general, LGG tend to have more resident microglial TAMs, while the HGG have the highest proportion of infiltrating monocytic TAMs (18).

##### 2.1.1.1 Myeloid cells in pLGG

The TME in pLGG can promote tumor growth by various interactions between the neoplastic cells and the microglia, T-cells and chemokines, while mechanisms such as oncogene-induced senescence, which results from MAPK/ERK hyperactivation, can result in growth arrest (3).

PA related to neurofibromatosis type 1 (NF1) contain significantly higher percentages of microglia compared to sporadic PA, suggesting a role for a microglia-tumor interplay driven by NF1 related gliomagenesis. It was shown in mice that microglia cells harbor a growth-promoting role in glioma maintenance, and that the ablation of microglia during optic glioma development reduces tumor proliferation. Furthermore, *Nf1* heterozygosity resulted in spatial and temporal differences in the amount of microglia present (19).

Additionally, Guo et al. studied tumor growth of NF1 optic glioma stem cells and found that the combined effects of T-cells and microglia infiltration by the optic glioma stem cells (o-GSC) were responsible for differences in the production of Ccl5, resulting in differences in growth pattern of the tumor (20). Before, it was already shown that the activation of T-cells results in the secretion of soluble factors that stimulate microglia in mouse brain to express Ccl5, providing a supportive TME for o-GCS engraftment (21).

In LGG, differences in myeloid cells have been observed based on the BRAF-status of the tumor. *KIAA1549::BRAF*-fusions occur in 30-40% of LGG (22), and these tumors show an enrichment of M1 (pro-inflammatory)-macrophages and M2 (pro-regenerative) -microglia, compared with BRAF^WT^ tumors. Also, while the tumors with BRAF-fusions promoted M2-microglia, the BRAF-V600E tumors promoted the enrichment of M2-polarized macrophages (23). In *KIAA1549:BRAF* fused pilocytic astrocytoma, Ccl2 is important for the recruitment of microglia and establishing a permissive TME for tumor growth (24).

##### 2.1.1.2 Myeloid cells in pHGG

In the previously mentioned study by Grabovska et al, a large group of pHGG previously described (5), was analyzed using methylCIBERSORT. Here, the number of monocytes varied according to the subgroup of pHGG. Especially in tumors with H3 G34 mutations, there was a higher level of immune cell infiltration, with higher levels of monocytes. Additionally, the pHGG with MAPK mutations had higher immune infiltrates, mostly comprised of monocytes and CD4+ T-cells (14).

Another study also identified differences according to the subtype of GBM. Here, in pGBM, the mesenchymal subtype showed an enrichment of gene signatures that are associated with microglia/macrophages and monocytes. In another similar analysis, including astrocytoma from grade II to IV, there was a significant enrichment of the M2-macrophage gene signature. In this cohort, there was an enrichment of macrophage/microglia, monocyte and granulocyte cell signatures in adult GBM patients with a short survival, but no specific immune cell signature could be identified in pGBM tumors with the shortest survival (25).

In the study by Griesinger et al., it was shown that when comparing LGG and HGG, PA had the most activated myeloid phenotype based on the expression of HLA-DR and CD64. GBM, on the other hand, had a more suppressive M2 phenotype, with higher expression of the M2-markers CD163 and CD206 (26). PA show lower amounts of CD68+ macrophages compared to GBM (27). Furthermore, the extent of infiltration of immunosuppressive CD163+ macrophages in pGBM, is not related to survival as opposed to adult GBM (28). Regarding the location, the hemispheric WT pHGG had more M1-macrophages compared to brainstem H3 K27M pHGG (29).

Although diffuse intrinsic pontine glioma (DIPG) or diffuse midline glioma (DMG) are part of the pHGG group, these are of specific interest due to their unique location, mutations and abysmal prognosis. When Lieberman et al. studied the myeloid population, the median number of CD68+ macrophages and microglia was similar in both pLGG, pHGG and DIPG. However, the pLGG and pHGG had higher numbers of CD163+ macrophages, which was not observed in DIPG. This suggested that DIPG do not recruit TAM and do not repolarize TAM to an immunosuppressive phenotype (28). Lin et al. also found that microglia and macrophages in DIPG have a non-inflammatory phenotype that do not strictly fit into a classical M1- or M2- phenotype (30).

However, in the analysis of Ross et al. using Nanostring expression profiling, the DMG were found to have a greater inflammatory TME component compared to hemispheric pHGG. In further histological analysis there was a high variability between samples regarding the number of IBA+ TAMs, but there was no difference observed between the DMG and GBM samples. The IBA1 positivity here correlated strongly with the expression of CD31, PDGFRβ and PDGFB. Using immunocompetent mouse models, they showed that peripheral bone-marrow-derived macrophages (BMDM) are the predominant TAM population (and not the microglia) (13). Additionally, they also found CCL3 to be an important component in the recruitment of these TAMs, which could therefore be a possible target for treatment.

Studies in adult GBM identified glioma cancer stem cells to polarize macrophages/microglia to an immunosuppressive state, which can be partially reversed by blocking STAT3 (31). Furthermore, in transgenic GBM mice, the inhibition of CSF-1R blocked gliomagenesis and resulted in tumor debulking. This is again explained by the TAM which become anti-tumorigenic and develop a phagocytic phenotype. However, in these mouse models, resistance occurs and results in rebound tumors, caused by an aberrant PI3K signaling and clinical trials in adult glioma patients with monotherapy CSF-1R inhibition showed no significant improvement in PFS (32–34).

#### 2.1.2 NK-cells

NK-cells are part of the innate immune system and function as cytotoxic effector cells (35). In PA, CD56+ NK cells are virtually absent compared to GBM (27). In a TCGA study, LGG with high expression of the Killer cell lectin-like receptor (KLR) family (encoding NKG2 NK-cell receptors), was associated with an improved prognosis (36). In the subtypes of pHGG, more NK-cells have been observed in hemispheric WT pHGG compared to brainstem H3 K27M pHGG (29). In another study, however, no significant difference was found in the infiltration of NK-cells between pHGG and normal control tissue (35).

#### 2.1.3 Eosinophils

Eosinophils are another understudied cell type in glioma, with only few studies present. In the study of Grabovska et al., eosinophils were found to contribute up to 13% of non-cancer cells in the tumor; and this tends to collide with the types of brain tumors that have high numbers of Tregs and NK-cells. The number of eosinophils present was negatively associated with tumor grade and there were significantly less eosinophils in G34-mutated GBM (14). Regarding the location of the tumors, significant differences were identified between WT hemispheric pHGG and K27M brainstem pHGG, with more eosinophils in the hemispheric tumors (29).

#### 2.1.4 Neutrophils

In an early study by Fossati et al., the level of infiltrating neutrophils correlated with tumor grade (37). Other studies confirmed this finding and showed that neutrophils promote the proliferation of glioma stem cells. Additionally, the neutrophils in the tumor possibly promote angiogenesis by inducing a proneural to mesenchymal subtype transition, through the upregulation of S100A4 (38). In pHGG, neutrophil infiltration was shown to be a negative prognostic factor for both hemispheric and brainstem locations (29).

In adult GBM patients, the pretreatment neutrophil-to-lymphocyte ratio (NLR) correlated with OS, with a high pre-treatment NLR correlating with a high neutrophil infiltration and low T-cell infiltration in the tumors, which predicted a poor OS (39).

### 2.2 Cells from the adaptive immune system in the TME

#### 2.2.1 T-cells

T-cells are one of the better studied cell types in the TME. In general, the lower grade tumors have a higher T-cell infiltration compared to higher grade tumors (26, 40). But even within the lower grade tumors, the levels of infiltration can be highly variable (40). For instance, the study by Robinson et al. identified higher T-cell infiltration and -density in pleomorphic xanthoastrocytoma (PXA) and ganglioglioma (GG), even though the study population was small. These two groups also had higher proportions of T-cell clusters (defined as T-cells with more than 10 other T-cells close to them in a 30 µm radius) compared with pilocytic astrocytoma (PA) or pHGG, which is an interesting finding, since PXA and GG are generally low in mutational burden. Additionally, the presence of the stem cell marker SOX2 in the tumor cells was inversely correlated with the T-cell infiltration and TCF1+ T-cells were enriched in perivascular areas. Tang et al. also studied the TME of PXA and GG using methylCIBERSORT, and observed that they exhibit lymphocytic infiltration, with significant increases of CD8+ T-cells in PXA, and suggested that this could be used to enhance the effects of immunotherapy (41). While they observed significant increases in CD8+ T-cells in PXA, no significant differences were seen in CD14+ macrophages, CD19+ B-cells, endothelial cells or fibroblasts (41). In a TCGA study of LGG, enrichment of memory CD8+ T-cells was associated with a better prognosis (36).

Considering pHGG, Lin et al. found that the TME in DIPG is rather non-inflammatory, with low levels of infiltrating T-cells, combined with low to absent expression of chemo- and cytokines (30). The location and type of the underlying driver mutation in pHGG also seem to influence the type and number of T-cells present. For instance, more CD8+ T-cells can be found in hemispheric H3 WT pHGG compared to H3 K27M-mutated pHGG located in the brainstem (29). This was also found in the study by Lieberman et al., where increased numbers of CD8+ T-cell infiltration in pLGG and pHGG were observed in comparison to control tissue, but not seen in DIPG (28). Histone H3-mutant glioblastoma are depleted of lymphocytic clusters compared to IDH-mutant and WT glioma. This compares to the immunologically quiet immune subtypes as described for LGG (42).

T-helper cells (CD4+) are important in the mediation of the anti-tumor immunity of the CD8+ cytotoxic T-cells. In the study of Grabovska et al., the infiltration of T-helper cells decreased with increasing WHO grade. Additionally, in H3 G34-mutant pHGG, higher than median concentrations of T-helper cells were associated with poor OS (14).

Additionally, the TME in glioma can have hypoxic regions and is low in nutrients, which can negatively impact the function of the T-cells, resulting in T-cell suppression, reduced memory T-cell survival and inhibition of T-cell proliferation (43).

An exception to the fact that pediatric glioma are generally cold tumors, are the tumors arising in children harboring germline biallelic mismatch repair deficiency (MMRD). In these patients, there is the potential to use immune checkpoint inhibition (ICB), while the efficacy of ICB is lower in the non-MMRD tumors, based on a more limited amount of tumor-infiltrating lymphocytes (44). It was shown that a high CD8+T-cell infiltration in tumors with MMRD + PPD (polymerase proofreading deficiency) predicted response and an improved OS (45). However, a large study investigating adult glioma with acquired MMRD after temozolomide treatment, observed that the hypermutation results in subclonal alterations which do not generate optimal antitumor T-cell responses. Both the MMR-deficient and the MMR-proficient glioma had a significant lack of T-cell infiltrates (in this study compared to MMR-deficient colorectal carcinoma) (46).

Little is known about the spatial organization of the TME in pediatric glioma. Pediatric HGG showed the most positive CD3- and CD8-staining around the perivascular spaces and necrotic areas (13), a phenomenon that was not observed in PA (27). In a study by Schaettler et al., the immunogenic profile of spatially distinct regions was studied from both glioma and brain metastases in adult patients. Glioma were mutationally more heterogeneous and subclonal and the intratumoral TCR repertoire was more heterogeneous in spatially distinct regions with locally expanded T-cell clones. In their study, they did not observe significant intratumoral differences in the immune infiltrate or activation, despite the heterogeneity in the variants and neoantigens within glioma based on bulk RNA-sequencing (47). Another study in adult glioma showed that T-cells infiltrating in GBM had high expression of immune checkpoints (PD-1, LAG-3, TIM3 or TIGIT), which were correlated with a loss of effector function (48). It remains to be determined whether the same principles apply to pediatric tumors.

##### 2.2.1.1 Regulatory T-cells

Regulatory T-cells (Tregs, CD4+ FOXP3+) play a role in the homeostasis of the immune system by suppressing abnormal immune responses. They can inhibit antitumor immunity and thus play a role in the development and progression of tumors (49). Tregs are increased in pediatric glioma and have been identified as a potential target for therapy (50). In the study of Heimberger et al. which included both adult and pediatric tumors, the number of Tregs was variable according to the tumor grade, with the highest numbers of Tregs in the higher grade astrocytoma. No significant differences were found between the newly diagnosed and recurrent tumors. Additionally, Tregs were found more in astrocytoma than oligodendrogliomas (51).

Furthermore, differences can be found in the subtypes of pHGG, with lower levels of Tregs in G34 R/V hemispheric pHGG, compared with their WT counterparts. Also, the hemispheric WT tumors harbor more Tregs than K27M brainstem tumors (29).

##### 2.2.1.2 NK T-cells

Another specific subtype of T cells, namely NK T-cells recognize lipid antigens presented by non-classical class Ib MHC CD1d antigen-resenting molecules. These are other possible actors in tumor immunity, which can be specifically interesting since the brain is high in lipids. However, their function in brain tumors and the few studies performed did not provide clear answers regarding their role in brain tumor biology (52).

#### 2.2.2 B-cells

B-cells are involved in the adaptive immune system and are responsible for antigen presentation and antibody production. Their role in tumor immunity is still somewhat controversial, and can vary among tumor types (53).

In comparison with previously discussed cell types, B-cells have not been studied intensively. As mentioned before, differences were observed concerning the number of T-cells present in PXA and GG, but in this study, no significant differences in the number of B-cells were observed (41). In pHGG, differences were observed, based on the underlying molecular alterations of the tumor. In the hemispheric WT pHGG, memory B-cells, compared with CD4+ regulatory T-cells and activated dendritic cells had a positive prognostic impact (29). G34-mutated tumors had an increased B-cell gene expression prevalence (42).

Another study in adult glioma identified B-cells to influence the other cells of the TME. These B-cells differentiate into regulatory B-cells (Bregs) under the influence of PlGF (placental growth factor)-carrying exosomes, that in turn suppress the activity of CD8+ T-cells (54).

## 3 The neural microenvironment in pediatric glioma

Normal neurons and astrocytes in and surrounding the tumor, can also influence the growth of the tumor. As shown by Venkatesh et al., neuronal activity caused an increase in the proliferation of HGG through the synaptic adhesion molecule neuroligin-3 (NLGN3), which promotes the proliferation of the cells through the PI3K-mTOR pathway, but also through the Src and Ras pathways (55). Furthermore, the dependencies of HGG on NLGN3 render this molecule a possible therapeutic target and by using ADAM10-inhibitors, which prevent the release of NLGN3, the growth of HGG in xenografts was reduced (56). Besides NLGN3, brain-derived neurotrophic factor (BDNF) and 78-kDa glucose-regulated protein (GRP78) were also identified as possible activity-regulated glioma mitogens (55).Another study by the same group, showed that the synaptic and electrical integration of glioma cells into neural circuits promotes the progression of these cells. A depolarizing current in an electrical network caused glioma progression in HGG (57).

The role of neurons and astrocytes has been studied in more detail in adult GBM. In a study by Heiland et al., tumor-associated astrocytes had an activation of the JAK/STAT pathway and showed expression of CD274 (PD-L1). The astrocytes showed a reactive state with high levels of IL10 and IFNγ, which results in an activation of the JAK/STAT pathway (58). Another study in genetically engineered mouse models of adult GBM, suggest that hypoxic astrocytes play a role in the growth of the tumor and could maintain stemness. Moreover, the extracellular matrix produced by these astrocytes under the influences of hypoxia can increase the proliferation and the drug efflux capabilities of tumor cells (59). When astrocytes go into a reactive state, they display gene signatures that are similar to a mesenchymal (MES) GBM subtype. Furthermore, the MES transition in adult GBM is associated with an induction of reactive astrocyte gene sets (60). Glial cells play a role in the phenotypic “go-or-grow” switch in adult GBM, and these effects are associated with the activation of the ERK, AKT and JNK signaling pathways (61).

## 4 Implications of the tumor microenvironment for therapy

### 4.1 Conventional therapy

#### 4.1.1 Chemotherapy

Dexamethasone, a commonly used corticosteroid, is frequently used in patients with brain tumors for its anti-inflammatory function and to treat brain swelling due to the tumor or treatment. However, it is known for its toxicity on immune cells, which can in turn have effects on the composition and function of the TME. Furthermore, it influences the blood-brain barrier (BBB), closing the latter and possibly resulting in a lower tissue penetration of systemic therapies (62).

Temozolomide (TMZ), a commonly used alkylating agent which excellent brain penetration, can cause lymphopenia and is also used for lymphodepletion prior to the use of T-cell-based immunotherapies (43). TMZ has an immunomodulatory effect, such as the enhancement of antigen-specific T-cell proliferation (63). It induces reactive astrogliosis, similar to RT and is discussed later on (59).

Regarding other types of chemotherapy, little is known on their effects on the TME. Several large studies, such as the HIT-GBM studies (64, 65), have been conducted, however these only investigated responses and toxicities and not the effects on the TME. Preclinical studies using co-cultures of glioma cells and astrocytes treated with TMZ and vincristine, show evidence of a chemoprotective effect based on direct contact between astrocytes and glioma cells (66). Other preclinical work showed that there was an increased chemoresistance in adult GBM for cisplatin, TMZ and etoposide in hypoxic conditions (67).

#### 4.1.2 Monoclonal antibodies

Anti-VEGF therapy (bevacizumab), was initially designed to affect brain tumor vascularization and has been described to reduce brain edema by improving the structure of the blood-brain-barrier (68), and as such can also affect the influx of inflammatory cells towards the tumor (69). Bevacizumab did not improve event-free survival (EFS) in pediatric patients with newly diagnosed HGG when added to standard radiotherapy and temozolomide treatment (HERBY trial) (70). When further analyzing these cases on a molecular and pathological level, however, the presence of high levels of CD8+ T-cells in the central tumor area was significantly associated with a better OS when receiving bevacizumab in addition to the standard RT/TMZ treatment (71). Some tumors acquire resistance to the treatment, which can be due to an increased recruitment of neutrophils during this treatment, promoting glioma progression. This could be due to S100A4, and upon depletion of S100A4, anti-VEGF therapy in mice bearing glioma was more efficacious (38). Upon anti-VEGF treatment, a correlation was seen between the decrease in tumor vascularity and reduction in myeloid cell infiltration in orthotopic glioma (72). Another study in adult GBM patients also indicated that only patients with a high peripheral neutrophil count benefited from treatment with bevacizumab (73).

#### 4.1.3 Radiotherapy

Radiotherapy (RT) is frequently used in the treatment of pediatric brain tumors, but has various side effects. Due to the possible neurocognitive side effects, RT is generally avoided in patients younger than 5 years. Furthermore, in patients with underlying neurofibromatosis type 1 (NF1), RT is generally avoided since they have an increased risk for secondary malignancies or cerebral vasculopathy (74, 75).

RT induces lethal DNA damage to the cells of the tumor and its surroundings. It results in the release of tumor-associated antigens, which then activate antigen-presenting cells (APC). These migrate to the draining lymph node where cytotoxic T-lymphocytes are primed, resulting in an adaptive immune response (76). Furthermore, radiotherapy can induce immunogenic cell death at the lesions borders, caused by the danger-associated molecular patterns (DAMP) that recruit immune cells and an increase in MHC1 expression (77). Therefore, RT can increase the tumor immunogenicity, turning a “cold” TME into a “hot” TME and thus sensitizing them for immunotherapy (77, 78). However, one has to consider that it is sometimes necessary to associate dexamethasone to treat the side effects of RT, which could theoretically counteract the tumor immunogenicity.

RT also induces changes in astrocytes, inducing reactive astrocytes, which promote the stemness in GBM, which could possibly contribute to recurrence in a later stage. Astrocyte-derived transglutaminase 2 (TGM2) has been identified as a potential radiation-induced modifier of the TME in adult GBM, protecting against cell death through radiation (79).

In other cancer types, the combined use of radiation therapy and inhibition of TGFβ (using neutralizing antibodies), resulted in a T-cell mediated rejection of the irradiated tumor and non-irradiated metastases in mice models (i.e. generating an ‘in situ’ tumor vaccine). Additionally, an improved outcome was seen when this was combined with PD-1 blockade (80).

Furthermore, radiotherapy could also induce an immunosuppressive state in the TME on the long term. Fibroblast attracted to the tumor (cancer-associated fibroblasts, CAF), can influence the immune cells and tumor progression through the release of cytokines and growth factors (81).

The classically used treatment options for pediatric glioma thus influence the TME in several ways and can have opposite effects. Additionally, the ‘side’ effects of these treatments provide other therapeutic options.

#### 4.1.4 Others – drugs under investigation

Besides the classic treatment options, a lot of research is ongoing to improve the PFS and OS of pediatric glioma patients. Of course, these novel treatments can also influence the TME. Keane et al. showed that the pHGG cells can polarize microglia towards a tumor-supportive phenotype, a phenomenon that could be reverted by reducing *Ezh2* gene expression in the microglia using EZH-inhibitors, which caused a decrease in the global H3K27me3 expression levels, and promoted an activation state in the microglial cells, resulting in antitumoral properties (82). Yin et al. had similar findings using co-culturing models where EZH2 was suppressed, resulting in an increase of M1-markers and an increase in the phagocytic capacities of microglia (83).

The mTOR pathway is another promising treatment option. In the study of Hsu et al., the combined use of rapamycin and hydroxychloroquine influenced the polarization of the TAMs from an M2 to an M1-phenotype. In such context, ICB could be more beneficial (84).

HDAC8-inhibition using the specific inhibitor PCI-34051 causes an enhanced activity of NK-cell *in vitro*, and increases in the infiltration of NKG2D+ and CD69+ NK-cells *in vivo*. Additionally, HDAC8-inhibition inhibits chemotaxis of microglia in the tumor *in vitro*. Further *in vivo* data shows that HDAC8-inhibition stimulates the expression of pro-inflammatory genes in TAM, indicating that HDAC8 on itself drives the TAMs to an anti-inflammatory phenotype (85).

Furthermore, research is ongoing on the use of nanoparticles in the treatment of pediatric glioma. These might offer a better drug delivery to the tumor and lower possible side-effects. Additionally, nanoparticles also stimulate the immune system and thus influence the TME in these tumors, however, their specific effects have not been studied in pediatric glioma (86).

### 4.2 Implications for therapy: immunotherapy

Immunotherapy has emerged as a novel paradigm in cancer treatment over the last years. The efficacy of immunomodulating strategies has been proposed to depend on the presence of cytotoxic immune cells in the tumor or in the peripheral blood, which could migrate to and eradicate the tumor. An important hallmark is, however, that these cytotoxic cells need to be stimulated and become active. The low number of infiltrating T-lymphocytes and thus the immunologically ‘cold’ status of the tumors, implies a poor response to immune checkpoint inhibition (87). For instance, the TME of DIPG is not highly immunosuppressive, but it lacks the effector immune cells (28). In pediatric patients, one also has to consider that the immune system might not be fully mature yet and is age-dependent (11), which might have an influence on tumor progression and the response to immunotherapy. Additionally, Bailey et al. suggested that immunotherapeutic options for pHGG will have to consider the location of the tumor. They suggest that the pHGG located in the hemispheres might respond well to vaccines, immune checkpoint blockade (ICB) and depletion of macrophages. On the other hand, brainstem tumors would benefit more from adoptive cell therapies, epigenetic modulation and new surgical delivery techniques (29). Several immunotherapy studies are currently ongoing in pediatric brain tumor patients (88). **Table 2** provides an overview of the current active immunotherapy studies in pediatric glioma. The majority of these studies are phase 1 trials.

**Table 2:** Immunotherapy.

Trial number	Treatment	Target	Disease	Phase
Immune checkpoint blockade				
NCT02359565	Pembrolizumab	PD-1	Recurrent, Progressive or Refractory DIPG, Non-Brainstem HGG, Ependymoma, Medulloblastoma or Hypermutated Brain Tumors	Phase 1
NCT01952769	Pidilizumab	PD-1	DIPG	Phase 1 – phase 2
Vaccine therapy				
NCT03988283	Personalized neoantigen DNA vaccine	/	Pediatric Recurrent Brain Tumor	Phase 1
NCT03914768	DC vaccination	/	DIPG or glioblastoma	Phase 1
NCT04978727	SurVaxM	survivin	progressive or relapsed medulloblastoma, HGG, ependymoma and newly diagnosed DIPG.	
NCT03396575	TTRNA-DC vaccines with GM-CSF	/	DIPG, Brain Stem Glioma	Phase 1
NCT04911621	DC vaccination	WT1	HGG, DIPG	Phase 1 – phase 2
NCT02960230	H3.3 K27M Peptide Vaccine with Nivolumab	H3.3 K27M	DIPG; DMG, H3 K27M-Mutant	Phase 1 – phase 2
NCT04943848	rHSC-DIPGVax	Neo-antigen heat shock protein	DIPG; DMG, H3 K27M-Mutant	Phase 1
NCT04749641	Histone H3.3-K27M Neoantigen Vaccine Therapy	H3.3 K27M	DIPG	Phase 1
NCT03299309	PEP-CMV	pp65	recurrent medulloblastoma and malignant glioma	Phase 1
NCT05096481	PEP-CMV	pp65	Newly diagnosed HGG or DIPG, recurrent medulloblastoma	Phase 2
CAR-T cell therapy				
NCT03638167	EGFR806-specific CART cell	EGFR806	EGFR-positive recurrent or refractory pediatric CNS Tumors	Phase 1
NCT04099797	(C7R)-GD2.CART cells	GD2	GD2-expressing Brain Tumors	Phase 1
NCT03500991	HER2-specific CART cells	HER2	HER2 positive recurrent/refractory pediatric central nervous system tumors	Phase 1
NCT04510051	IL13Rα2 CART cells	IL13Rα2	Recurrent malignant primary brain tumors	Phase 1
NCT04185038	B7-H3-Specific CAR T Cells	B7-H3	DIPG, DMG and recurrent or refractory CNS tumors.	Phase 1
NCT05768880	SC-CAR4BRAIN	B7-H3, EGFR806, HER2, IL13-zetakine	DIPG, DMG, and recurrent or refractory CNS tumors.	Phase 1
Virotherapy				
NCT05717712	Ad-TD-nsIL12	/	DIPG	Phase 1
NCT02457845	HSV G207	/	Recurrent supratentorial brain tumors	Phase 1
NCT03911388	HSV G207	/	recurrent or refractory cerebellar brain tumors	Phase 1
NCT04758533	AloCELYVIR	/	DIPG, recurrent medulloblastoma	Phase 1 - phase2
NCT05717699	Ad-TD-nsIL12	/	progressive DIPG	Phase 1
Others				
NCT04837547	Tumor-specific ex vivo expanded autologous lymphocyte transfer (TTRNA-xALT)	/	newly diagnosed DIPG or recurrent neuroblastoma	Phase 1
NCT05887882	Universal Donor TGFβi Natural Killer Cells	/	Recurrent or progressive supratentorial malignant brain tumors	Phase 1

Active trials in immunotherapy (including those not yet recruiting). Completed, terminated or withdrawn trials were omitted from this overview.

#### 4.2.1 Immune checkpoint blockade

Both PD-1 and PD-L1 are frequent targets in ICB. Programmed death ligand 1 (PD-L1; B7-H3; CD274) is the ligand of programmed death receptor 1 (PD-1) and in normal situations, plays a role in the maintenance of immune cell tolerance (89).

The efficacy of ICB has been studied in children, however with unfavorable success. This has been associated with the low mutational burden in these tumors, the low expression of major histocompatibility complex and the high prevalence of macrophages in the TME (44, 45).

The exception here seems to be hypermutant tumors, as already discussed before, driven by mutations leading to mismatch repair deficiency (MMRD) (44). Children with constitutional MMRD develop several types of tumors and have a poor prognosis, frequently not reaching adulthood (90). Recently, a trial was conducted where pediatric patients with MMRD and/or polymerase-proofreading deficiency (PPD) tumors were treated with PD-1 inhibitors (pembrolizumab/nivolumab). Here, an OS of 39.3% and a progression-free survival of 26.9% was reported for recurrent/progressive CNS tumors, which is a major improvement compared to their standard (unsuccessful) treatment. An improved survival upon ICB could be seen in tumors with high levels of single nucleotide variants (SNVs) and total MS-indels. They also discovered that clonal mutations were strong predictors of survival and response. Furthermore, a high CD8-T cell infiltration was predictive for response and improvement of survival, while no association between CD68 with response or survival could be identified. After all, the pediatric MMRD+PPD gliomas responded better to ICB compared to their adult counterparts (45).

Bockmayr et al. identified within the mesenchymal subgroups of HGG, two immunologically infiltrated clusters, where an increased gene expression indicative of a higher infiltration of CTL in one of the subgroups could be used for ICB. Additionally, they showed that in glioma which were K27-mutated, PD-L1 and CTLA4 correlated with a worse prognosis. This was the opposite for G34-mutated tumors, which also seem to rely more on TGFB1 and HAVCR2 for their immune escape. Therefore, ICB targeting the PD-1/PD-L1 axis could be beneficial for patients with DMG with H3 K27M mutations (42), while targeting HAVCR2/TIM3 could be an alternative approach (91).

TGFB has been suggested to play a role in the resistance to PD-L1 therapy, since it would be contributing to the exclusion of T-cells in the tumor. TGFB would have an inhibitory effect on the cytotoxic activity of T-cells and would promote the generation of regulatory T-cells (92).

A recent clinical study investigated the effect of MDV9300 (pidilizumab) in patients with DIPG (NCT01952769), which is an immune-modulating IgG1 monoclonal antibody targeting the NOTCH ligand DLL1 and to a lesser extent PD-1 (93, 94), which seems to enhance endogenous antitumor hematologic malignancies. Although a small study, the preliminary results implicated a tolerable safety profile and suggests some clinical activity (94).

#### 4.2.2 Tumor neoantigen vaccine therapy

Vaccine therapy comprises several types of therapy, such as dendritic cell vaccination, vaccination with tumor lysate or peptide vaccination with glioma-associated antigens. These vaccines could possible synergize with CAR T-cell therapy (43).

In DC vaccination therapies in DMG, the lack of immune cells may hamper its efficacy, suggesting that strategies to enhance immune cell trafficking should potentially be combined (95).

The heterogeneity in tumors can cause difficulties for the use of vaccines since not all tumor cells express the same tumor antigens, resulting in the escape of some subclones of the tumor to treatment. This might be overcome by the use of vaccines that target multiple neoantigens.

Pollack et al. investigated the use of glioma-associated antigen (GAA) epitopes in a peptide vaccination study in children with HGG and recurrent LGG, showing preliminary evidence of immunological and clinical response. However, no assessments on the changes in the TME itself were performed (96, 97).

The H3 27M in pHGG has been identified as a possible target in vaccine strategies. It was shown that that the H3 K27M can act as an immunogenic neoepitope, and is targetable by a specific peptide vaccine. In a humanized mouse model, it resulted in IFNγ immune responses, induced by both CTLs and Th1-cells (98). Others developed a high-affinity TCR that recognizes an H3.3K27M epitope which is expressed by H3.3K27M+HLA-A*0201+ glioma cells. This finding has been used to start a vaccine trial with the H3.3K27M26-35 epitope peptide in children with H3.3K27M+ DIPG or HGG (PNOC-007, NCT02960230) (99). However, the study is still enrolling patients and no results have been described so far.

#### 4.2.3 CAR T-cell therapy

Chimeric antigen receptor (CAR) T-cell therapy is based on the immune system of the patient itself, where the immune cells of the patient are engineered to target a tumor-selective epitope. Theoretically, this could result in more specific targeting of the tumoral cells, while the normal tissue (which does not express the neo-antigen) could be spared. Additionally, targeting tumors with CARs is not dependent on MHC-antigen presentation. This could be beneficial in those tumors with a low tumor mutation burden or with defects in antigen presentation (43). CAR T-cell therapy has been used before in B-cell malignancies, with good clinical activity (100). However, in adult brain tumors, this therapy had variable results, where a lot of the determinants are still incompletely understood (43). For pediatric brain tumors, several targets are being evaluated, with several still in clinical trials (101).

Haydar et al. investigated 49 patient-derived orthotopic xenograft models of pediatric brain tumors and established a rank order of expression for 5 potential targets (B7-H3, GD2, EphA2, IL-13Ra2 and HER2), and found that B7-H3 and GD2 were highest in expression (102). Trials with CAR T-cells against B7-H3 (CD276) are still ongoing, but some preclinical data shows possible benefit from this therapy (103). Anti-GD2 CAR T-cells with an incorporated 4-1BBz costimulatory domain, demonstrated a robust antigen-dependent cytokine generation and DMG cell killing *in vitro* (104). In some of the first phase I clinical trials in a limited patient group (n=4), which only recently published its first results, the anti-GD2 CAR T-cells showed promising results on the tumor volume, although not without therapy-related side effects (105).

Importantly, it was observed that the expression of these target antigens is heterogeneous, illustrating the need to target multiple antigens, to avoid an immune escape (102). Additionally, several reports found that there can be a loss or decrease in the target antigen expression, implying that in the further development of this therapy for brain tumors, additional strategies will be necessary to minimize or avoid antigen escape (43).

The TME of the tumors can influence the effect of CAR T-cell therapy, where suppressive monocyte-derived TAMs could influence the effector functions and microglia-derived TAMs are needed to promote the effector functions of the T-cells (101). Tregs could also potentially influence the efficacy of CAR T-cells, where they can augment the growth of the tumor and suppress the T-cell effector molecules in the TME of glioma. This can result in possible negative effects on the function of the CAR T-cell (101). The CAR T-cells on the other hand can also induce changes in the TME. For example, an increased production of INFγ caused by the CAR T’s, can induce immunostimulatory effects and inhibit the macrophages to differentiate to a M2-phenotype (78).

The use of combination therapies was also proposed, such as the addition of PD-L1 inhibition, together with CAR T-cell therapy. This might overcome the immunosuppressive TME, and therefore also improve the persistence and functioning of the CAR T-cells. Clinical phase I studies (in adults, NCT03726515) are currently being performed but no results have been reported yet (43).

#### 4.2.4 Oncolytic virotherapy

Oncolytic virotherapy delivers a live but modified virus that specifically targets tumor cells. The advantage of this type of therapy is that the tumor cells are directly targeted or that the host immune system is activated to attack the tumor cells by stimulating the effector function and antitumor activity of T-cells in the TME. Oncolytic viruses have also been suggested to be used in combination with CAR T-cells, since these viruses can be engineered to express transgenes which can target the suppressive TME (43).

A recent pilot study showed an increased influx of CTLs, combined with a greater proportion of macrophages and microglia after treatment with oncolytic HSV G207 therapy in a pediatric patient with pGBM. This would suggest that oHSV therapy would increase or amplify the anti-tumor immune response (106). Additionally, these T-cells also expressed higher levels of checkpoint proteins such as CTLA-4, PD-1, PD-L1 and IDO after treatment (106).

Tejada et al. describe a clinical phase I trial in which the oncolytic virus DNX-2401 (formerly Delta-24-RGD) is administered intratumorally to children with newly diagnosed DIPG. Preliminary results indicate that this therapy is well tolerated, but longer follow-up and enrolling additional patients to further assess efficacy and safety is still needed (107).

Mendez et al. generated an endogenous mouse model of ACVR1-mutant brainstem glioma and tested the effects of immune stimulatory gene therapy using an adenovirus that expressed thymidine kinase and fms-like tyrosine kinase 3 ligand (Flt3L). The adenoviral delivery of the TK/Flt3L caused an anti-tumor immunity with the recruitment of anti-tumor specific T cells, which even resulted in an increased median survival. The treatment with TK/Flt3L resulted in an increase in tumor-specific CD8+ T-cells and an increased toxicity (enhanced IFNγ production) (108). Before, research of the same group had already shown the efficacy of an immune stimulatory gene treatment in rat and mouse models of adult GBM, leading to tumor regression and long term survival, and went further on to a clinical trial in adult GBM patients [NCT01811992 (109)] (110, 111). Other types of gene therapy for glioma fall beyond the scope of this review and are described elsewhere (112).

#### 4.2.5 NK-cell therapy

Recently, an engineered human NK-cell therapy for adult glioblastoma was developed, with NK-cells having multi-CARs to target GD2 and NKG2D. In addition, an antibody fragment that blocks the activity of CD73 is released via the cleavage of a tumor-specific linker, dependent on proteases in the TME. However, this is still preclinical data, based on studies in a patient-derived xenograft model of adult GBM (113). Shortly, the NCT05887882 trial (phase 1) will start in pediatric glioma patients, concerning the intratumoral injection of ex vivo expanded NK cells (see **Table 2**).

## 5 Conclusion and future directions

Over recent years, the tumor micro-environment of brain tumors gained more interest and has been the subject of multiple studies. However, these studies frequently investigated only a subset of cells present in the TME and were mainly focused on tumors occurring in adult patients, focusing on adult GBM. Only more recently, one realized that pediatric brain tumors are a separate cohort, where the TME has its own characteristics. Although more studies have been published in recent years, it appears that these cannot yet provide a complete overview of the TME in itself. The information is still scattered and can be contradicting.

The treatment and thus survival of patients with brain tumors has been lagging, with only few successes over the past decades. Many of the trials and new therapies were focused on the tumor cells, while it has been shown that the TME has an effect not only on tumor growth but is also affected by the treatment itself. With the success of immune checkpoint blockade therapy in other cancer types, it was hoped that similar results would be obtained in brain tumors. However, the expected results were not forthcoming, except for tumors occurring in patients with MMRD. Novel therapies acting on the TME, such as tumor vaccination and CAR T-cell therapy showed some promising results in (pre)-clinical trials, but are not established therapies in the treatment of pediatric brain tumors. Large studies with a sufficient number of patients are still lacking, and questions on efficacy, feasibility and toxicities still remain. Furthermore, questions remain on the possible options of combining these treatments and on the ideal route of delivery to the tumor.

In this review, we aimed at providing a comprehensive overview of what is known about the TME of pediatric glioma and how it is both affected by therapy and used as a therapeutic option. Despite many research efforts, it is clear that many knowledge gaps still exist and that further investigation is still necessary, both on a fundamental level to understand the TME and the interactions between the cells in the TME and in the setting of therapy and clinical trials. Not only do we need studies in larger groups of patients, illustrating the need for international consortia, but also extended investigational methods such as bulk and single cell sequencing and spatial omics techniques enabling the in-depth study and comparison of the TME in pediatric glioma.

## 6 Methods

Articles were searched using Pubmed on 15^th^ of February 2022. The following search terms were used: “child”, “pediatric”, “glioma”, “tumor micro-environment”. The search retrieved a total of 2185 articles. These were imported into the online tool Rayyan to select the articles further on used in this review. Duplicates were first removed. Afterwards the articles were categorized in the categories ‘include’, ‘maybe’, ‘exclude’.

Articles focusing specific on pediatric types of tumors were first studied, after which others were included. References from the included articles were studied and possible interesting studies were thereafter also included.

The information regarding the clinical trials was retrieved via the ClinicalTrials.gov website (accessed on the 3^rd^ of July 2023).

The figure was created with BioRender.com.

## Author contributions

JM: conceptualization, investigation, writing-original draft. SJ: conceptualization, writing-review & editing, supervision. FS: conceptualization, writing-review & editing, supervision. All authors contributed to the article and approved the submitted version.

## Glossary

**Table:**

APC	antigen presenting cell
BBB	blood brain barrier
BMDM	bone marrow derived macrophage
CAR	chimeric antigen receptor
DAMP	damage-associated molecular pattern
DC	dendritic cell
DIPG	diffuse intrinsic pontine glioma
DMG	diffuse midline glioma
EFS	event-free survival
ERK	extracellular signal-regulated kinase
EZH2	enhancer of zeste homolog 2
GAA	glioma-associated antigen
GBM	glioblastoma
GG	ganglioglioma
HDAC	histone deacetylase
HSV	herpes simplex virus
ICB	immune checkpoint blockade
IDH1	isocitrate dehydrogenase 1
INFγ	interferon gamma
MAPK	mitogen-activated protein kinase
mgTAM	microglial tumor-associated macrophage
MHC	major histocompatibility complex
MMRD	mismatch repair deficiency
moTAM	monocytic tumor-associated macrophage
mTOR	mammalian target of rapamycin
NF1	neurofibromatosis type 1
NLGN3	neuroligin-3
NK cell	natural killer cell
NLR	neutrophil to lymphocyte ratio
NOTCH	Neurogenic locus notch homolog protein
OS	overall survival
PA	pilocytic astrocytoma
PD-1	programmed cell death protein 1
PD-L1	programmed death ligand 1
PDX	patient-derived xenograft
PFS	progression-free survival
pHGG	pediatric high-grade glioma
pLGG	pediatric low-grade glioma
PPD	polymerase proofreading deficiency
PXA	pleiomorphic xantoastrocytoma
RT	radiotherapy
SNV	single nucleotide variant
TAM	tumor-associated macrophage
TCR	T-cell receptor
TME	tumor micro-environment
TMZ	temozolomide
VEGF	vascular endothelial growth factor
WT	wild-type
